# Supplementary material for: Genotoxic formaldehyde and lipid aldehydes are sources of DNA damage in keratinocytes
Source: bioRxiv. 2025 Nov 14:2025.11.13.688345. Preprint. [Version 1] doi: 10.1101/2025.11.13.688345 (PMC12642467; doi:10.1101/2025.11.13.688345)
Supplement: 1 [file NIHPP2025.11.13.688345V1-supplement-1.pdf]

bioRxiv preprint doi: https://doi.org/10.1101/2025.11.13.688345; this version posted November 14, 2025. The copyright holder for this preprint (which was not certified by peer review) is the author/funder, who has granted bioRxiv a license to display the preprint in perpetuity. It is made available under aCC-BY 4.0 International license.

Supplemental Data

Genotoxic formaldehyde and lipid aldehydes are sources of DNA damage in keratinocytes.

Nicolas J. Blobel[1]*, Yibing Yao[1]*, Marian C. Okondo[1], Agata Smogorzewska[1]

Affiliations:

[1]Laboratory of Genome Maintenance, The Rockefeller University; New York, NY, 10065, USA.

*These authors equally contributed
Correspondence: Agata Smogorzewska (asmogorzewska@rockefeller.edu)

Supplemental data include:

Figs. S1 to S5
Table S1 Sequences of sgRNAs used in the study
Table S2 Plasmids used in this study
Table S3 (Excel): Keratinocyte RNA-Seq results.

bioRxiv preprint doi: https://doi.org/10.1101/2025.11.13.688345; this version posted November 14, 2025. The copyright holder for this preprint (which was not certified by peer review) is the author/funder, who has granted bioRxiv a license to display the preprint in perpetuity. It is made available under aCC-BY 4.0 International license.

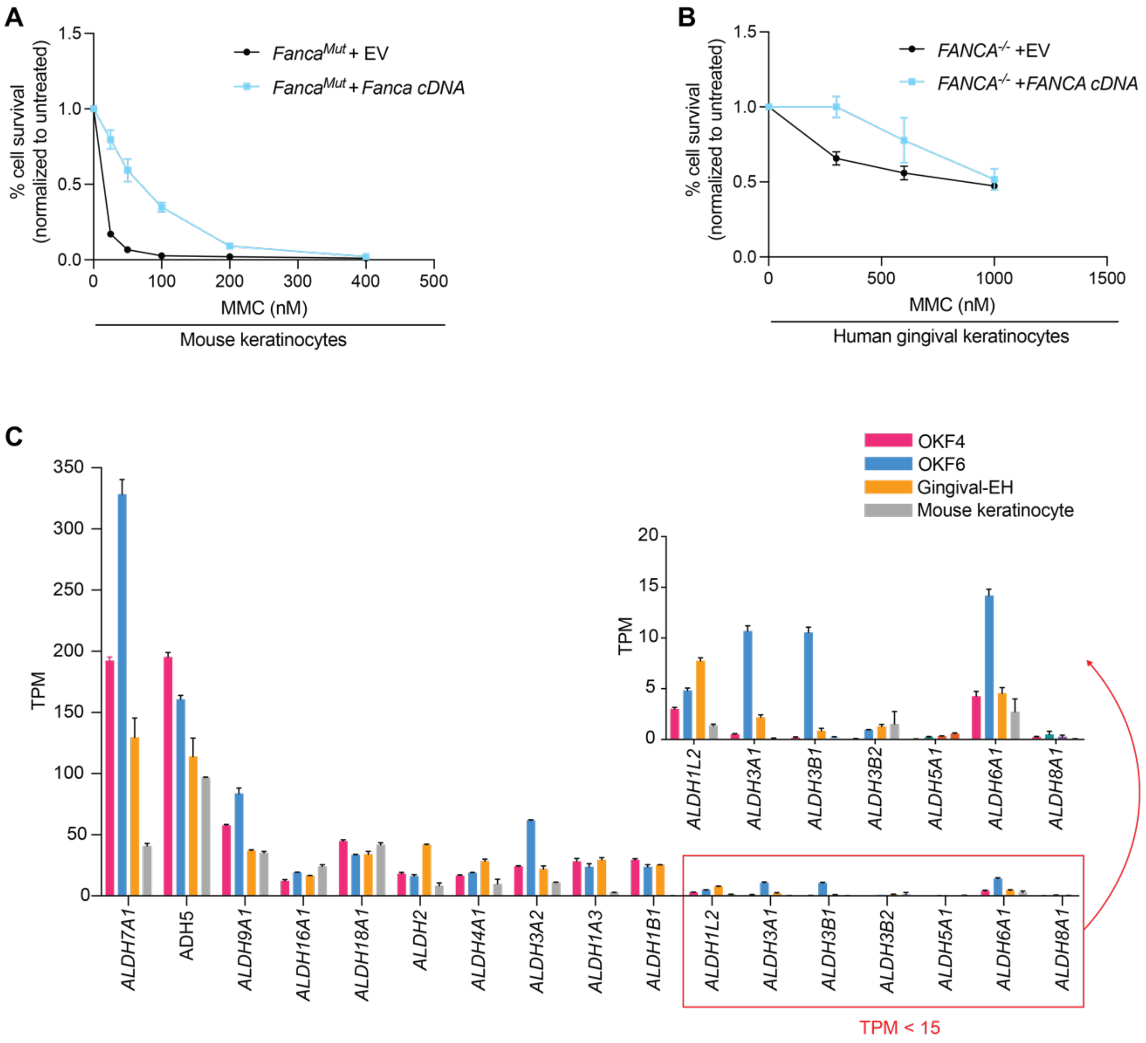

ALDH gene with no detectable expression (<1TPM): *ALDH1A1, ALDH1A2, ALDH1L1, ALDH5A1* and *ALDH8A1*
ADH genes with no detectable expression (<1TPM): *ADH1A, ADH1B, ADH1C, ADH4, ADH6, ADH7* and *ADHFE1*

Supplemental Figure 1. Further characterization of keratinocyte cell lines from human and mouse.

(A) MMC sensitivity assay in mouse keratinocytes. FA-deficient (*Fanca*^{Mut}) keratinocytes were complemented with empty vector (EV) or *Fanca* cDNA. Cells were treated with indicated dose of MMC and then cultured for 5 days before cells were counted to calculate percent of cell survival.

(B) MMC sensitivity assay in human *FANCA*$^{-/-}$ gingival-Eh cells complemented with EV and *FANCA* cDNA. Experiment was carried out as in (A).

(C) RNAseq expression levels of all *ADH* and *ALDH* family genes in the OKF4, OKF6, gingival-Eh, and mouse keratinocyte lines. On the top right, a zoomed-in plot shows genes with low expression (TPM<15). Genes with no detectable expression (TPM<1) are summarized at the bottom. See Table S3.

bioRxiv preprint doi: https://doi.org/10.1101/2025.11.13.688345; this version posted November 14, 2025. The copyright holder for this preprint (which was not certified by peer review) is the author/funder, who has granted bioRxiv a license to display the preprint in perpetuity. It is made available under aCC-BY 4.0 International license.

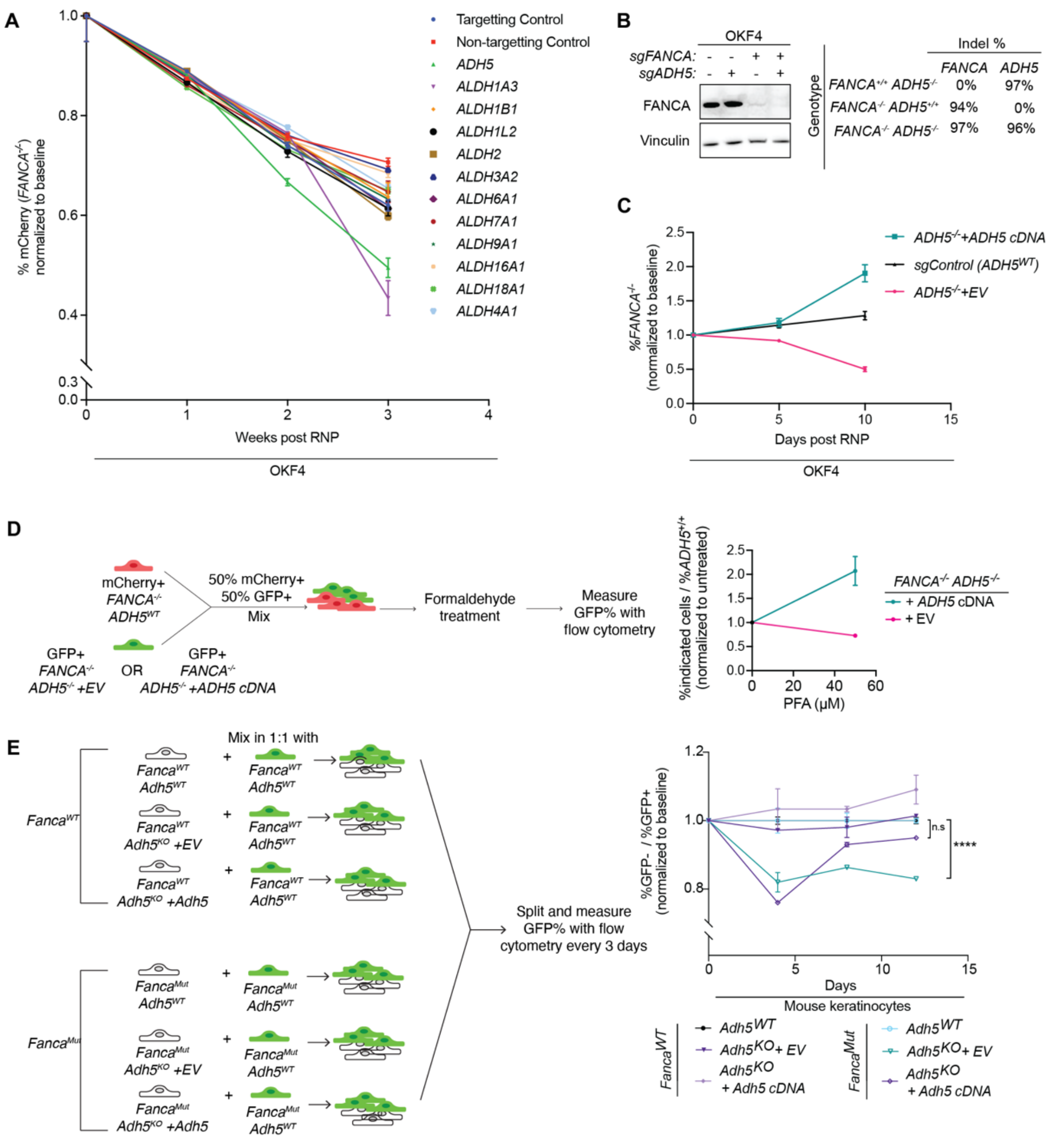

Supplemental Figure 2. The loss of ADH5 leads to a growth defect in FA-deficient keratinocytes and sensitizes them to formaldehyde.

(A) Time course of the percent of FA-deficient cells over the duration of the competition assay shown in Figure 2B for the single-gene knockouts of *ADH* or *ALDH* in OKF4.

(B) Validation of *FANCA* and *ADH5* knockout in OKF4 cells. *Left*: Immunoblotting of FANCA in OKF4 cells with CRISPR-Cas9 knockouts with indicated sgRNAs. Vinculin was used as a loading control. *Right*: Summary of the percent of insertion and deletion (indel) at each gene locus.

(C) Time course of the percentage of FA-deficient cells corresponding to the competition assay shown in Figure 2D.

(D) Sensitivity to PFA in OKF4 *ADH5* knockout cells complemented with either empty vector (EV) or *ADH5* cDNA, measured by competition assay. *Left*: Schematic of competition assay setup. Cells were treated with indicated dose of PFA and cultured for 3 days before they were analyzed by flow cytometry. *Right*: The ratio of *FANCA*$^{-/-}$ *ADH5*$^{-/-}$ over *FANCA*$^{-/-}$ at each dose was normalized to the untreated control. Data are presented as mean ± SD.

(E) Competition assay in mouse keratinocytes with *Adh5* knockout as indicated in the schematic (left). Time course of the percentage of GFP- cells over the duration of the experiment corresponding to Figure 2E.

bioRxiv preprint doi: https://doi.org/10.1101/2025.11.13.688345; this version posted November 14, 2025. The copyright holder for this preprint (which was not certified by peer review) is the author/funder, who has granted bioRxiv a license to display the preprint in perpetuity. It is made available under aCC-BY 4.0 International license.

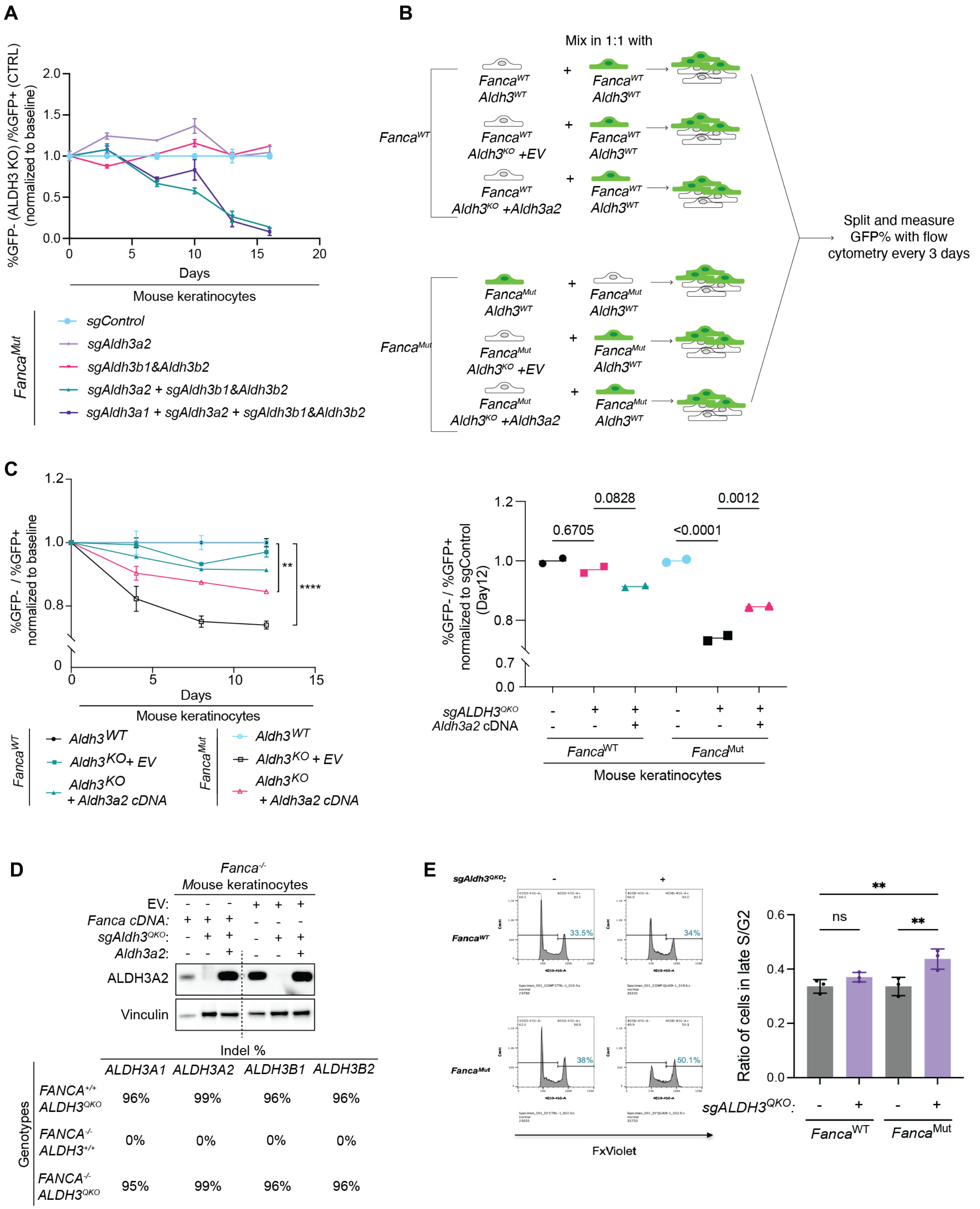

Genotypes	ALDH3A1	ALDH3A2	ALDH3B1	ALDH3B2
FANCA+/+ ALDH3QKO	96%	99%	96%	96%
FANCA-/- ALDH3+/+	0%	0%	0%	0%
FANCA-/- ALDH3QKO	95%	99%	96%	96%

Supplemental Figure 3. The loss of ALDH3 family genes leads to a growth defect in FA-deficient keratinocytes and sensitizes them to treatment with inducer of lipid peroxidation.

(A) Time course of the percent of GFP- cells corresponding to the competition assay in Figure 3D.

bioRxiv preprint doi: https://doi.org/10.1101/2025.11.13.688345; this version posted November 14, 2025. The copyright holder for this preprint (which was not certified by peer review) is the author/funder, who has granted bioRxiv a license to display the preprint in perpetuity. It is made available under aCC-BY 4.0 International license.

(B) Schematic of competition assay setup for mouse keratinocytes with *Aldh3* quadruple knockout.
(C) Competition assay in mouse keratinocytes with *Aldh3* quadruple knockout in FA-competent ($Fanca^{WT}$) or FA-deficient ($Fanca^{Mut}$) backgrounds. For each sample, the ratio of %GFP- over %GFP+ cells after 12 days of culture was normalized to the starting ratio at the beginning of experiment and then to sgControl. *Left*: Time course of the percent of GFP- cells corresponding to the competition assay shown in Supp Figure 3B. Each dot represents one technical replicate, and data are presented as mean ± SD. *Right*: Quantification of competition assay after 12 days of culture. Each dot represents one technical replicate, and statistical significance was evaluated by one-way Kruskal-Wallis ANOVA
(D) Validation of *Aldh3* quadruple knockout in mouse keratinocytes. *Top*: Immunoblotting of ALDH3A2 in mouse keratinocytes with CRISPR-Cas9 knockouts with the indicated sgRNAs. Vinculin was used as a loading control. *Bottom*: Summary of the percentage of insertion and deletion (indel) at each gene locus.
(E) Cell cycle analysis in *Aldh3* quadruple knockouts in FA-competent and FA-deficient mouse keratinocytes. *Left*: Representative FxViolet DNA content histograms. *Right*: Quantification of percentage of cells in late S/G2 phase. Each dot represents one technical replicate, and statistical significance was evaluated by one-way Kruskal-Wallis ANOVA.

bioRxiv preprint doi: https://doi.org/10.1101/2025.11.13.688345; this version posted November 14, 2025. The copyright holder for this preprint (which was not certified by peer review) is the author/funder, who has granted bioRxiv a license to display the preprint in perpetuity. It is made available under aCC-BY 4.0 International license.

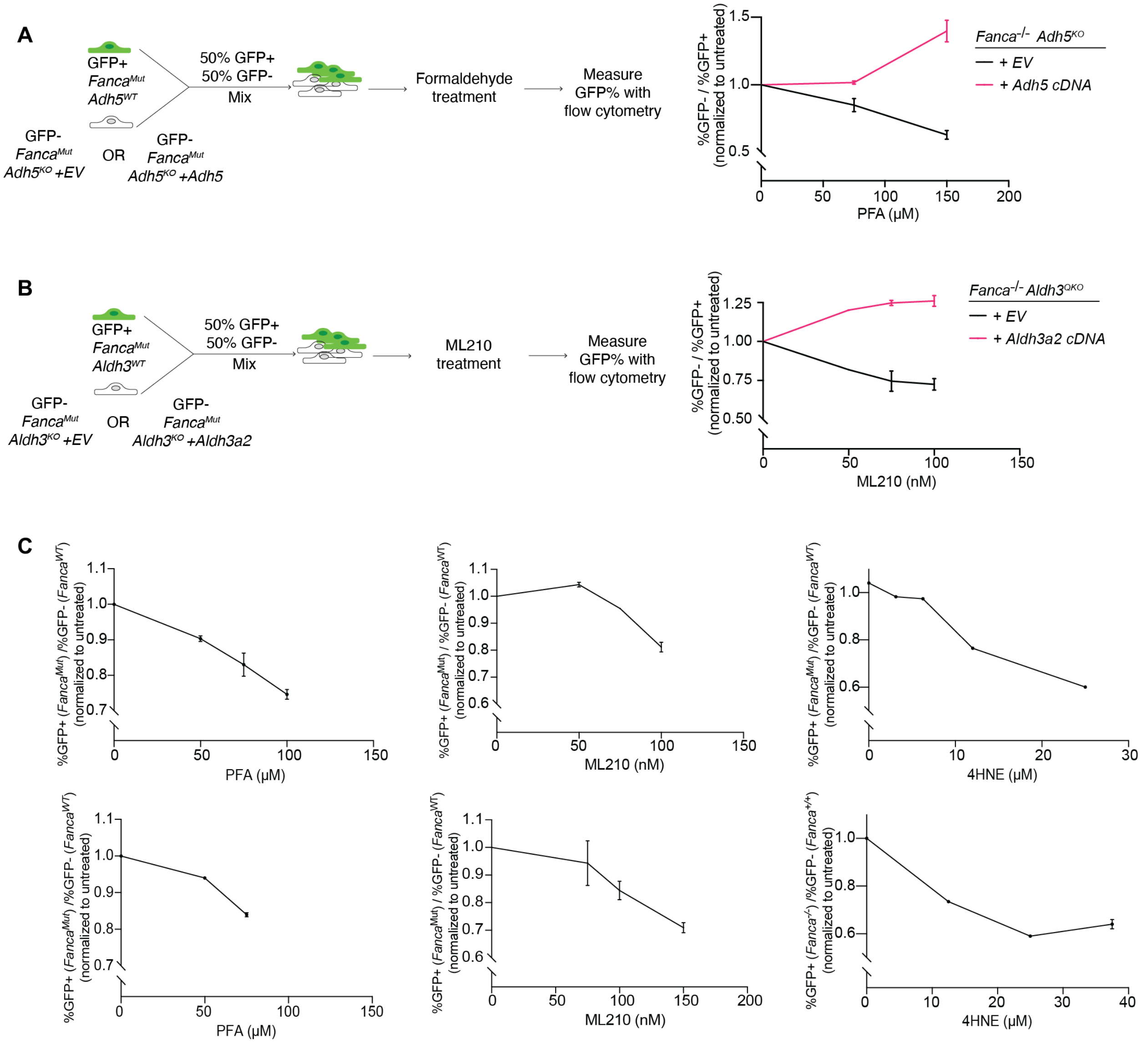

Supplemental Figure 4. FA-deficient mouse keratinocytes exhibit sensitivity to multiple inducers of ICLs and ferroptosis.

(A) Sensitivity to PFA in mouse keratinocyte *Adh5* knockout complemented with either empty vector (EV) or *Adh5* cDNA, measured by competition assay. *Left*: Schematic of competition assay setup. Cells were cultured with indicated dose of PFA and cultured for 3 days before they were analyzed by flow cytometry. *Right*: Ratio of percentage of GFP- over percentage of GFP+ cells were normalized to the untreated control. Data are presented as mean ± SD.

(B) Sensitivity to ML210 in mouse keratinocyte *Aldh3* quadruple knockout complemented with either empty vector (EV) or *Aldh3a2* cDNA, measured by competition assay. *Left*: Schematic of competition assay setup. Cells were treated with indicated dose of ML210 and cultured for 3 days before they were analyzed by flow cytometry. *Right*: Ratio of percentage of GFP- over percentage of GFP+ cells were normalized to the untreated Data are presented as mean ± SD.

(C) Replicate competition assays assessing the sensitivity of FA-deficient mouse keratinocytes treated with increasing doses of PFA, ML210, or 4HNE, compared to FA-competent controls. Cells were treated and cultured for 3 days prior to flow cytometry analysis. *Fanca*^{Mut} cells exhibited increased sensitivity to PFA, ML210, and 4HNE.

bioRxiv preprint doi: https://doi.org/10.1101/2025.11.13.688345; this version posted November 14, 2025. The copyright holder for this preprint (which was not certified by peer review) is the author/funder, who has granted bioRxiv a license to display the preprint in perpetuity. It is made available under aCC-BY 4.0 International license.

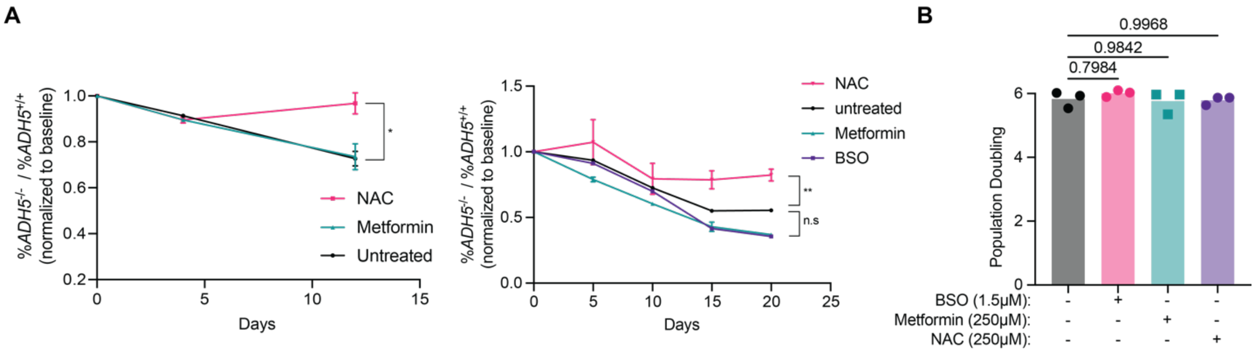

Supplemental Figure 5. The growth defect of FA-deficient *ADH5*$^{-/-}$ OKF4 is partially rescued with supplementation of NAC, but not metformin.

(A) Competition assays assessing the growth of FA-deficient *ADH5*$^{-/-}$ compared to FA-deficient *ADH5*$^{+/+}$ OKF4 cells, treated with the indicated doses of NAC, metformin or BSO. Cells were cultured for the indicated number of days, and treated once daily with 250μM NAC, 250μM metformin, and 1.5μM BSO. Only NAC treatment partially rescued the dropout of FA-deficient *ADH5*$^{-/-}$ OKF4 cells.

(B) Growth of *FANCA*$^{-/-}$ cells measured by cumulative population doublings over 8 days with daily treatment of NAC, metformin, and BSO.

bioRxiv preprint doi: https://doi.org/10.1101/2025.11.13.688345; this version posted November 14, 2025. The copyright holder for this preprint (which was not certified by peer review) is the author/funder, who has granted bioRxiv a license to display the preprint in perpetuity. It is made available under aCC-BY 4.0 International license.

Table S1. Sequences of sgRNAs

Gene	Species	Target gene and exon	sgRNA Sequences
Control	Human	Targeting intergenic region	(1) GTAATACAGACCCATCTCCC<br>(2) GCGGATTAGAGGTAATGCGG
	Mouse		GTTCTTGATTAAGTTAAGAC
FANCA	Human	Exon 4	CACGCTAGAGGCAACCATCC
TP53	Human	Exon 4	AACCATTGTTGAATATCGTC
ADH5	Human	Exon 5	ACTTTATCCAAAGGTGCTAA
	Mouse	Exon 6	AAGACGGCACAAGTAGAACC
ALDH1A3	Human	Exon 1	TTGACCTCCAGGTTGCGGAT
ALDH1B1	Human	Exon 2	CCGACGGTCAACCCTACCAC
ALDH1L2	Human	Exon 2	AAAGAGGGCCACCGAGTAGT
ALDH2	Human	Exon 2	CGTCAATCCGTCCACTGGAG
ALDH3 family (quadruple knockout)	Human	*ALDH3A1* exon 5<br>*ALDH3A2* exon 3<br>*ALDH3B1* exon 7<br>*ALDH3B2* exon 7	(1) TTCGACCATATCCTGTACAC<br>(2) GTGAGCACGTTCTTCTTAAC<br>(3) ACTGTTTCTGGTTGATGATG
	Mouse	*Aldh3a1* exon 4<br>*Aldh3a2* exon 4<br>*Aldh3b1* exon 7<br>*Aldh3b2* exon 7	(1) TGAATGGACCTCCTACTACG<br>(2) ATAATCAGTACGACTCCCAG<br>(3) GCTACACAGGATATAATCGG
ALDH4A1	Human	Exon 6	ACCAACAGCACGGTGTACCG
ALDH6A1	Human	Exon 4	GATAGCGGAGCAAGACCTGC
ALDH7A1	Human	Exon 4	CGGGAGAAGATCCAAGTACT
ALDH9A1	Human	Exon 4	GTATAACCAAACGATCCACC
ALDH16A1	Human	Exon 1	CACCTCGCTGGAGTACGGAC
ALDH18A1	Human	Exon 2	GACGGTTGTACACTTGACCC

Table S2. Plasmids used in this study

Name	Source
pDONOR223	Invitrogen
pDONOR223-empty vector	Smogorzewska Lab
LentiCRISPRv2GFP (LCV2-GFP)	Addgene #82416
LentiCRISPRv2mCherry (LCV2-mCherry)	Addgene #99154
VSV-G; retroviral packaging	Smogorzewska Lab
Gag/Pol; retroviral packaging	Smogorzewska Lab
pMD2.G; lentiviral packaging	Addgene #12259
psPAX2; lentiviral packaging	Addgene #12260
pWZL-MSCV-hTERT	Smogorzewska Lab
pMSCV-Neo-HPV16 E6E7	Smogorzewska Lab

bioRxiv preprint doi: https://doi.org/10.1101/2025.11.13.688345; this version posted November 14, 2025. The copyright holder for this preprint (which was not certified by peer review) is the author/funder, who has granted bioRxiv a license to display the preprint in perpetuity. It is made available under aCC-BY 4.0 International license.

pMSCVhyg-*Fanca*	Smogorzewska Lab
pMSCVhyg-FANCA	Smogorzewska Lab
pMSCVneo-*Adh5*	Smogorzewska Lab
pMSCVneo-*ADH5*	Smogorzewska Lab
pMSCVneo-*Aldh3a25*	Smogorzewska Lab
pMSCVneo-*ALDH3A2*	Smogorzewska Lab
pLV-eGFP	Addgene #36083
pLV-mCherry	Addgene #36084
